# Genetic Diversity of Type A Influenza Viruses Found in Swine Herds in Northwestern Poland from 2017 to 2019: The One Health Perspective

**DOI:** 10.3390/v15091893

**Published:** 2023-09-07

**Authors:** Lukasz Rabalski, Maciej Kosinski, Piotr Cybulski, Tomasz Stadejek, Krzysztof Lepek

**Affiliations:** 1 Laboratory of Recombinant Vaccines, Intercollegiate Faculty of Biotechnology, University of Gdansk, Abrahama 58, 80-307 Gdansk, Poland; 2 Biological Threats Identification and Countermeasure Center of the General Karol Kaczkowski Military Institute of Hygiene and Epidemiology, Lubelska 4 St, 24-100 Pulawy, Poland; 3 Goodvalley Agro S.A., Dworcowa 25, 77-320 Przechlewo, Poland; 4 Department of Pathology and Veterinary Diagnostic, Institute of Veterinary Medicine, Warsaw University of Life Sciences-SGGW, 02-776 Warsaw, Poland

**Keywords:** zoonosis, emerging diseases, influenza, epidemiology, NGS sequencing, surveillance

## Abstract

Influenza A viruses (IAV) are still a cause of concern for public health and veterinary services worldwide. With (−) RNA-segmented genome architecture, influenza viruses are prone to reassortment and can generate a great variety of strains, some capable of crossing interspecies barriers. Seasonal IAV strains continuously spread from humans to pigs, leading to multiple reassortation events with strains endemic to swine. Due to its high adaptability to humans, a reassortant strain based on “human-like” genes could potentially be a carrier of avian origin segments responsible for high virulence, and hence become the next pandemic strain with unseen pathogenicity. The rapid evolution of sequencing methods has provided a fast and cost-efficient way to assess the genetic diversity of IAV. In this study, we investigated the genetic diversity of swine influenza viruses (swIAVs) collected from Polish farms. A total of 376 samples were collected from 11 farms. The infection was confirmed in 112 cases. The isolates were subjected to next-generation sequencing (NGS), resulting in 93 full genome sequences. Phylogenetic analysis classified 59 isolates as genotype T (H1avN2g) and 34 isolates as genotype P (H1pdmN1pdm), all of which had an internal gene cassette (IGC) derived from the H1N1pdm09-like strain. These data are consistent with evolutionary trends in European swIAVs. The applied methodology proved to be useful in monitoring the genetic diversity of IAV at the human–animal interface.

## 1. Introduction

Influenza A virus (IAV) belongs to the Orthomyxoviridae family and is the main causative agent of human and animal flu. The severity and geographical spread of the disease depend primarily on the origin and genetic evolution of the virus. Seasonal strains circulating among certain hosts typically cause waves of incidence. When some slight changes are introduced to viral surface glycoproteins due to the accumulation of mutations, such strains may be a reason for local epidemics, whereas major changes, or even the replacement of these proteins, can potentially lead to a pandemic [1].

The difference in the peptide sequences of two surface glycoproteins, hemagglutinin (HA) and neuraminidase (NA), is the basis of IAV classification into subtypes. Today, 18 types of HA and 11 types of NA are recognized. The vast majority of subtypes circulate among wild waterfowl and constitute a natural reservoir of IAV [2]. In the course of viral evolution, several “spill-over” events followed by virus adaptation introduced the IAV into susceptible species such as marine mammals, bats, horses, ferrets, pigs, and humans, where certain adapted strains continue circulating and undergo selective environmental pressure. In the case of human infections, zoonosis is usually restricted to infected people and rarely develops into human-to-human transmitted disease, although it is possible in certain conditions and when it happens, it might be dangerous for the human population. [3].

To become a transmissible disease, novel IAVs must replicate efficiently in a new host, and for this reason, they must undergo some adaptive changes that will allow them to cross the host species barrier [4]. The genome architecture of IAV and the process of viral assembly facilitate these events [5]. Eight segments of negative-polarity RNA encode viral proteins that have a significant impact on the specificity of the host and the efficiency of viral RNA replication and evade the immune response of the host [6]. RNA-dependent RNA polymerase, which is responsible for the replication of the genetic material of the virus, is an enzyme without proofreading properties that leads to the formation of point mutations in each replication cycle [7]. It also occurs in segments coding for major viral antigens, causing the accumulation of changes. This so-called antigenic drift may result in peptide sequence alteration, which translates directly to structural changes in the surface proteins. If the changes are sufficiently large to avoid specific antibodies raised against these proteins, it may lead to an epidemic. The mutation frequency is so high that after a few years, we can observe changes in the major virus variants circulating worldwide [8]. Such differences necessitate continuous monitoring of the variability of the virus to design the most effective vaccine each year [9]. 

When various strains of the virus circulate in the same area, one host cell may be infected with two different viral strains. After possible reassortation, a completely new set of genome segments originating from two different viruses would be incorporated into a viral particle giving rise to a whole new virus. This interchange in genetic segments is known as an antigenic shift. When such reassortment occurs between strains of different species specificity, there is a chance for a new dangerous strain with a pandemic potential to emerge. It would become widespread in a naive population with no previous contact with this strain, and due to other adaptations or changes (e.g., in viral polymerase subunits and receptor site recognition), it could efficiently replicate in new hosts, becoming a threat to the whole population [10].

Pigs susceptible to infection with both “avian” and “human” strains are believed to play the role of a closed subtype-mixing environment. This allows co-infection and creates conditions for the rearrangement of the genetic segments of the virus [11].

The epidemiology of swine influenza A virus in Europe is complex and dynamic. Although only four major swIAV subtypes, H1N1, H3N2, H1N2, and H1N1pdm09, are the most prevalent, numerous constellations of internal genes with genes coding for HA and NA are possible. This abundance of genotypes is caused by different origins of RNA segments as a result of spillover or reassortment events in the past [12]. Until the end of the 1970s, a “classical swine” H1N1 from Eurasian lineage was the dominant strain of swIAV in European countries. In 1979, this “classical” strain was entirely replaced by the introduction of an antigenically distinct H1N1 strain of avian origin (H1avN1) that seemed to have a selective advantage over its predecessor [13]. The circulating H3N2 strain emerged after genomic reassortment, which took place in the early 1980s, between H1avN1 and human-like swine H3N2 viruses. The most recent reassortment event in 1994 in Great Britain gave rise to the “human seasonal” H1N2 (H1huN2) strain, derived from human seasonal H1N1, swine H1avN1, and “human like” swine H3N2 strains [13,14]. H1N1pdm09 of pandemic origin has been circulating in the swine population since 2009 [15]. All these strains circulate among swine populations in Europe, and their different combinations of swine, human seasonal, human pandemic, and avian genes contribute to a plethora of reassortants. Some of these supplanted or even replaced endemic strains found in Denmark, Germany, or Sweden (H1avN2dk, H1pdm09N2, H3huN2, and H1avN2) [16,17,18]. Similar events have been reported in Italy, Belgium, and France [19,20,21]. The high genetic variability of circulating strains and the growing number of passive and active surveillance programs have created a need for a uniform nomenclature for the classification of HA lineages and swIAV genotypes. In 2015, Watson et al. (2015) [22] proposed a complex genotype nomenclature for 23 combinations of genes (A-W) based on 290 swIAVs collected in 14 European countries between 2009 and 2013. Phylogenetic analysis was based on nine major swIAV lineages that were circulating worldwide. With additional genotypes identified by Henritzi et al. (2020) [23], this genotyping system is well-established and widely used. A new classification for H1 lineages was developed by Anderson et al. (2016) [24], who divided H1 into three main lineages: Classical swine lineage-1A (with H1pdm09), Human seasonal lineage-1B, and Eurasian avian lineage-1C. These two classification systems proved to be useful for organizing swIAV diversity.

Since the influenza pandemic in 2009, numerous cases of swine-to-human zoonotic influenza infections have been reported, as well as a growing number of swine infections with human strains of the virus [25,26,27,28,29,30,31,32,33]. These findings highlight the importance of research in the field of influenza interhost relationships. In this context, surveillance and genome sequence analysis will prove useful for understanding the molecular evolution dynamics of influenza. The data we present here are part of a project aimed at assessing influenza A virus subtype diversity in Poland. In this study, we analyzed influenza A virus subtype diversity among swine herds in northwestern Poland along with phylogeny based on HA, NA, and PB2 genome segments. We discuss the necessity of large-scale surveillance of the common human–animal interface and possible ways to raise awareness of the threat posed by the emergence of a new viral strain with pandemic potential.

## 2. Materials and Methods

### 2.1. Collection of Samples from Swine and Isolation of Viral RNA

Samples were collected from farms in the northwestern region of Poland from pigs of various ages exhibiting influenza-like symptoms. Nasal swabs were collected with FLOQSwabs^®^ (COPAN Diagnostics Inc., Murrieta, CA, USA) and placed in 3 mL of the Universal Transport Medium (UTM^TM^, COPAN Diagnostics Inc.). The specimens were transported to the laboratory in a portable refrigerator at 4–8 °C. In the laboratory, the swabs were vortexed for 10 s and centrifuged for 5 min at 1000 rpm. Aliquots of 1 mL were used immediately for viral RNA isolation or stored at 4–6 °C for no longer than 48 h. The rest of the medium was stored at −70 °C for further use as an inoculum for virus isolation from the cell culture. Viral RNA isolation was performed using high-load volume funnel columns NucleoSpin^®^ RNA Virus F Kit (Macherey-Nagel GmbH, Düren, Germany) according to the manufacturer’s instructions.

### 2.2. Confirmation of Influenza A Virus Infection in Collected Samples

All samples were initially tested for the presence of influenza A-type genetic material using the virotype^®^ Influenza A RT-PCR Kit (QIAGEN, Venlo, Netherlands). Viral RNA isolated from swabs, including a positive control, a negative control, and an internal control of β-actin mRNA, were subjected to the Real-Time PCR protocol according to the manufacturer’s instructions on the Light Cycler^®^ 480 System (ROCHE, Basel, Switzerland).

### 2.3. Virus Isolation from Madin-Darby Canine Kidney (MDCK) Cell Culture

Monolayers of MDCK cells (MERCK) were cultured in T-25 flasks in D-MEM growth media (Corning, [+] 4.5 g/L glucose, L-glutamine, [−] sodium pyruvate) supplemented with antibiotic/antimycotic solution (Invitrogen) and 10% fetal bovine serum (FBS). When the cell layer was 90% confluent, the growth medium was decanted, and the cells were washed three times with 6 mL of pure D-MEM containing 2 μg/mL of TPCK-trypsin. One milliliter of each specimen (swabs in UTM) was used to inoculate T-25 flasks. The control flask was inoculated with 1 mL of pure UTM^TM^. After one hour of incubation at 37 °C and 5% CO_2_, the inoculum was replaced with 6 mL of viral growth medium (D-MEM growth media with 2 μg/mL of TPCK-trypsin and without FBS). Each flask was inspected daily and cultured at 37 °C and 5% CO_2_ until a cytopathic effect (CPE) was observed, usually after 3–7 days. The influenza virus was harvested by collecting the supernatant supplemented with 0.5% glycerol for stabilization. If no CPE was detected after 10 days, blind passage was performed on a new T-25 flask 2 times before the sample was considered unable to isolate the virus from the specimen.

### 2.4. Nucleic Acid Extraction from Viral Isolates and Amplification of the Whole Influenza A Virus Genome Segments

RNA from the viral isolates was extracted using the RNeasy^®^ Mini Kit (QIAGEN) according to the manufacturer’s instructions. Extracted RNA was used for whole-genome amplification using the SuperScript^TM^ III One-Step RT-PCR System with Platinum^TM^ *Taq* High-Fidelity DNA Polymerase (Thermo Fisher Scientific, Waltham, MA, USA). Briefly, 10 μL of extracted RNA from each isolate was mixed with 25 μL of master mix (containing 0.4 mM dNTPs, 2.4 mM MgSO_4_), 1 μL of SuperScript^TM^ III RT/Platinium^TM^ *Taq* High-Fidelity Enzyme Mix, 3 μL of 5 μM mix of sense and antisense primers (sense: 5′-CTGGATACGCCAGCRAAAGCAGG-3′; antisense: 5′-GACCTGATGCGGAGTAGAAACAAGG-3′), and 11 μL of PCR grade water. The reactions were placed in a pre-heated ProFlex PCR System thermal cycler (Thermo Fisher Scientific, Waltham, MA, USA) and amplified as follows: cDNA synthesis and denaturation: 1 cycle at 45 °C for 30 min, 95 °C for 1 min; PCR amplification, 5 cycles at 95 °C for 15 s, 47 °C for 30 s, 68 °C for 3 min; 23 cycles at 95 °C for 15 s, 57 °C for 30 s, 68 °C for 3 min; final extension 1 cycle 68 °C for 7 min. The amplified genome was analyzed on a 1% agarose gel stained with SimplySafe dye (EURx, Gdansk, Poland).

### 2.5. Preparation of Sequencing Library

PCR products were purified after an enzymatic reaction on magnetic beads using Agencourt AMPure XP (Beckman Coulter, Brea, CA, USA) according to the manufacturer’s instructions, utilizing 96 deep-well microtiter plates. The concentration of DNA in the samples after purification was measured using a Quantus^TM^ Fluorometer (Promega, Madison, WI, USA) and the samples were diluted to a concentration of 0.2 ng/μL. Diluted samples were used to create a sequencing library using the Nextera XT DNA Library Prep Kit in 96-well plates (Illumina, San Diego, CA, USA). Briefly, genomic DNA in samples was tagmented; after the addition of indexes, all libraries (each sample is a library with a unique index combination) were amplified, amplified libraries were purified on Agencourt AMPure XP magnetic beads, and libraries were checked for the right library size distribution on Agilent 4150 TapeStation System (Agilent Technologies, Santa Clara, CA, USA). All libraries were normalized using a bead-based normalization method, pooled, and diluted to a loading concentration suitable for the MiniSeq System (Illumina, San Diego, CA, USA)).

### 2.6. Whole-Genome Sequencing Using MiniSeq Platform (Illumina), Followed by Bioinformatical and Phylogenetic Analysis of Gathered Data

Whole-genome sequencing was performed on the MiniSeq platform from Illumina, and raw read data were basecalled and demultiplexed by the sequencing platform. Individual samples, represented as FASTQ files, each containing paired reads of 150 nucleotides in length, underwent bioinformatic analysis through two avenues: An automated process via the INSaFLU platform (https://insaflu.insa.pt/ (accessed on 1 of February 2023)) and an in-house pipeline. Qualitative verification was performed using FastQC (v.0.11.5), which served as a preliminary step for both methods. The automated process yielded the output sequences and a subtype-specific influenza virus report. The in-house pipeline employed tools integrated within Geneious Prime (v.2022) (Dotmatics, Bishops Stortford, England)). For instance, BBDuk (v.38.84) was utilized for trimming, whereas normalization and error correction were performed using BBNorm (v.38.84). De novo assembly was accomplished through the assembly utility embedded within Geneious Prime, using a medium sensitivity option. The obtained contigs were subsequently compared using MAFFT (v.7), and, as necessary (such as a discrepancy at the nucleotide position or an incomplete contig sequence), reads were mapped to the consensus using Minimap2 (v.2.17), thereby establishing a final output sequence. The full sequences corresponding to the segments of the influenza virus were compared, establishing that there were five genetic variants present in all sequenced samples. Preliminary HA and NA subtype classifications were performed using multiplex PCR assays, as described in detail by Chiapponi et al. (2021) [19]. H1 clade classification was performed as described in Anderson et al. (2016) [24] using the swine H1 influenza classification tool available on http://www.fludb.org, accessed on 22 February 2022. For phylogenetic inference, the HA, NA, and PB2 sequences were extracted and aligned using MAFFT (v.7), following which maximum likelihood (ML) trees were calculated using RAxML (v.8.2). The correct substitution model and its parameters for inferred evolutionary history was chosen based on Bayesian Information Criterion and corrected Akaike Information Criterion using MEGAX. For phylogenetic analysis, the GTR GAMMA model was utilized, featuring rapid bootstrapping and a search for the best-scoring ML tree, validated by 1000 bootstrap replicates. For the segment-specific collection utilized for analyses, additional sequences were included, specifically relevant swIAVs representing major European lineages, human reference strains associated with “human-like” swIAVs, and recent Polish isolates. Sequences were retrieved from Genebank InfluenzaVirus Resource Database (https://www.ncbi.nlm.nih.gov/genomes/FLU/Database/nph-select.cgi?go=database (accessed on 4 of May 2023)), and EpiFlu™ Database (http://www.gisiaid.org (accessed on 4 of May 2023)). The obtained data were used for genotyping according to the method described by Watson et al. (2015) [22]. Sequences of all eight genome segments were used for phylogenetic inference. The origin of each analyzed segment was named by clustering with the reference strains. A list of reference strains together with accession numbers can be found in Supplementary Table S1.

## 3. Results

A total of 376 samples were collected from 11 farms. All farms were located in the northwestern region of Poland. Sampling was conducted between May 2017 and August 2019. RNA was isolated from all samples for subsequent confirmation of the presence of influenza A virus using a virotype^®^ Influenza A RT-PCR Kit. We were able to confirm influenza A virus infection in 112 of 376 (29.8%) samples collected from 5 of the 11 facilities (45.5%). The infection prevalence in the farms ranged from 42.8% to 55%, with the exception of one farm, where the prevalence level was 80%. To isolate the virus, all of the influenza-positive samples served as an inoculum for MDCK cells, which were then cultured to check whether CPE would occur. We observed a strong CPE in 92 cultures. In the next four cultures, CPE was observed after the first blind passage, and 16 cultures showed no signs of CPE. The virus was harvested from all 96 CPE-positive samples. Viral RNA was extracted from all obtained isolates and used for whole genome amplification of influenza A virus. We successfully amplified all eight segments of the viral genome from 93 isolates. The PCR products were then used to create a set of sequencing libraries, and 93 samples were sequenced using a MiniSeq NGS sequencing system from Illumina. After raw data analysis, we assembled 93 sets of influenza A virus full genome segment sequences. Using the INSaFLU algorithm, we assigned a subtype to all 93 isolates. Thirty-four isolates were recognized as the A/H1N1 subtype and 59 as the A/H1N2 subtype. Isolates were further identified and classified as described by Chiapponi et al. (2021) [19]. Briefly, the HA and NA genes from each of the sequenced isolates were assigned to one of the HA lineage or NA subtypes, based on PCR analysis using linage and subtype-specific primers. Multiplex subtyping revealed that the HA gene from 59 of the isolates belonged to the H1-1C lineage, and all 59 corresponding NA genes were N2 subtypes. The remaining 34 isolates were classified as H1-1A and N1 subtypes, which matched the initial INSaFLU algorithm subtyping in full. Due to the high variability of HA segments between circulating strains, we decided to use a phylogeny-based global nomenclature system and an automated annotation tool for H1 hemagglutinin genes from swine influenza A viruses available online in the Influenza Research Database. The H1 clade classification showed that 59 isolates classified as HA-1C by multiplex PCR belonged to the 1.C.2 clade (63.4%) and were related to the Eurasian avian lineage, and the hemagglutinin from the rest of the isolates could be assigned to clade 1.A.3.3.2. (36.6%) of H1N1pdm09 origin. A summary of the sample collection process together with clade and lineage classifications for HA and NA subtypes is described in Table 1.

After multiple alignments of all segments, we found five different strains (I–V) representing all 93 isolates (three among H1N2 isolates and two in the group of H1N1 isolates), which we chose for further analysis. The names of the strains corresponding to the analyzed genetic variants and accession numbers to the full genome sequences submitted to the GISAID EpiFlu database are listed in Table 2.

**Figure 1:**
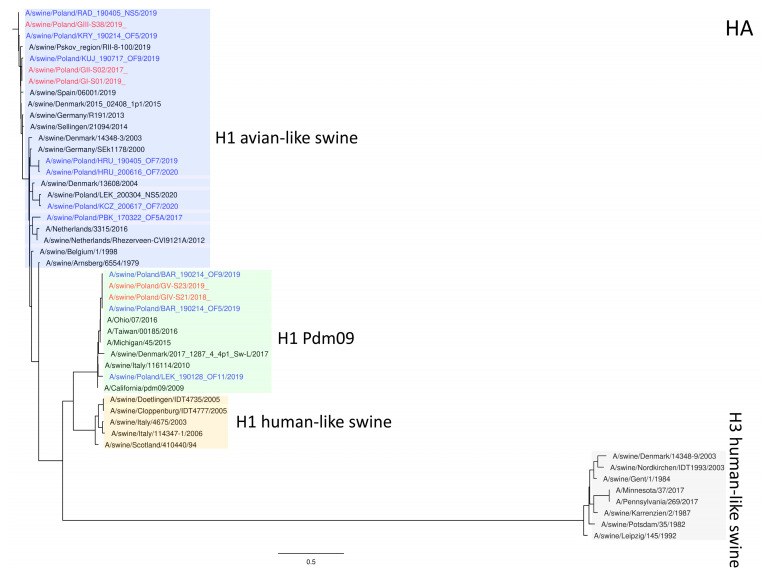
Maximum Likelihood (ML) phylogeny tree of segment HA of swine influenza viruses analyzed in this work. The tree was inferred using relevant swIAV strains representing major European lineages, human reference strains associated with “human-like” swIAVs, and recent Polish isolates. The reference dataset was retrieved from the GenBank Influenza Virus Resource Database and the GISAID EpiFlu™ database. The Polish isolate names from this study are marked in red, and recent Polish isolates are marked in blue. Known reference strains and those circulating in Europe are denoted in black. The major lineages are highlighted in color: H1 avian-like swine (Eurasian Avian), blue; H1 Pdm09 (Classical swine), green; H1 human-like swine (Human seasonal), orange; H3 human-like swine, beige. The tree was drawn to scale and validated by 1000 bootstrap replicates, with branch lengths indicative of the number of substitutions per site.

**Figure 2:**
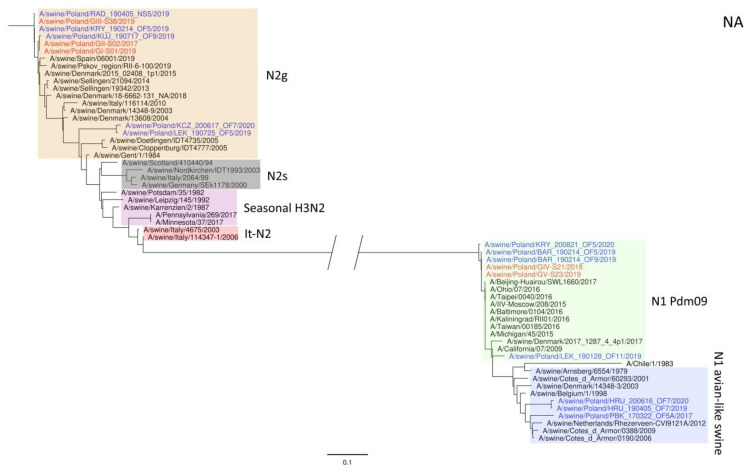
Maximum Likelihood (ML) phylogeny tree of segment NA of swine influenza viruses analyzed in this work. The tree was inferred using relevant swIAV strains representing major European lineages, human reference strains associated with “human-like” swIAVs, and recent Polish isolates. The reference dataset was retrieved from the GenBank Influenza Virus Resource Database and the GISAID EpiFlu™ database. The Polish isolate names from this study are marked in red, and recent Polish isolates are marked in blue. Known reference strains and those circulating in Europe are denoted in black. The major lineages are highlighted in color: N2g (A/swine/Gent/1/1984-like), orange; N2s (A/swine/Scotland/410440/94-like), gray; Seasonal H3N2, pink; It-N2 (A/swine/Italy/4675/2003-like), red; N1 Pdm09, green; N1 avian-like swine, blue. The tree was drawn to scale and validated by 1000 bootstrap replicates, with branch lengths indicative of the number of substitutions per site.

**Figure 3:**
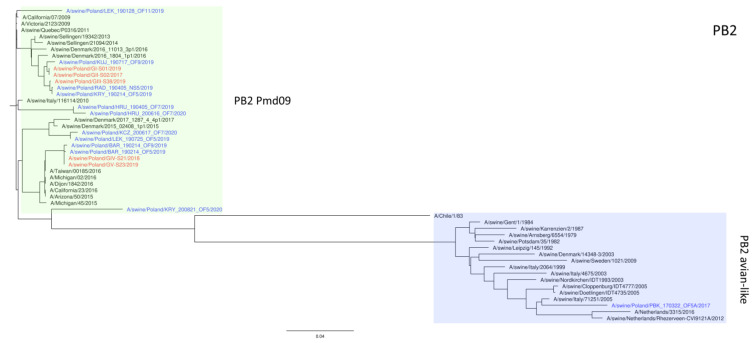
Maximum Likelihood (ML) phylogeny tree of segment PB2 of swine influenza viruses analyzed in this work. The tree was inferred using relevant swIAV strains representing major European lineages, human reference strains associated with “human-like” swIAVs, and recent Polish isolates. The reference dataset was retrieved from the GenBank Influenza Virus Resource Database and the GISAID EpiFlu™ database. The Polish isolate names from this study are marked in red, and recent Polish isolates are marked in blue. Known reference strains and those circulating in Europe are denoted in black. The major lineages are highlighted in color: PB2 Pdm09, green; Pb2 avian-like, blue. The tree was drawn to scale and validated by 1000 bootstrap replicates, with branch lengths indicative of the number of substitutions per site.

The phylogeny based on HA, NA, and PB2 segments revealed the relationship of the strains analyzed in this study to relevant swIAVs representing major lineages circulating in Europe and recent Polish isolates. As shown in Figure 1, three H1N2 strains clustered with swIAVs, which harbor the HA gene derived from the Eurasian avian lineage, while two H1N1 strains were closely related to human pandemic strains and swIAVs with the HA gene of pandemic origin. NA gene phylogeny (Figure 2) shows a clear division into H1N1 strains with A/California/07/2009-related clusters and H1N2 strains grouped with the A/swine/Gent/1/1984-like strains. When analyzing Figure 3, we can see that the PB2 segment from all analyzed strains originated from the H1N1pdm09 line. In all phylogenetic trees, the analyzed strains were located in the vicinity of recent Polish isolates classified as the corresponding genotypes.

To study the possible reassortment events, we applied the genotype classification system developed by Watson et al. (2015) [22]. The origin of internal gene cassette (IGC) was determined, and the results showed that IGCs from all strains are closely related to A/California/07/2009, therefore, H1N1 strains from this study can be assigned to a P genotype and all H1N2 strains are classified as genotype T (Table 3). Samples represented by strains A/swine/Poland/GIV-S21/2018(H1N1) and A/swine/Poland/GV-S23/2019(H1N1) were collected from the same farm 9 months apart and showed evidence of the circulation of the swIAV of the same genotype but were genetically different. The three H1N2 genetic variants, A/swine/Poland/GI-S01/2019(H1N2), A/swine/Poland/GII-S02/2017(H1N2), and A/swine/Poland/GIII-S38/2019(H1N2), represent different sampling locations and collection times.

## 4. Discussion

It is estimated that seasonal flu epidemics cause 2–5 million cases and 250,000–500,000 deaths worldwide annually [34]. The emergence of a new strain of influenza A virus in 2009 resulted in a pandemic that lasted over two years and was responsible for more than 60 million cases in the United States of America alone [8]. These numbers show that newly emerging strains of influenza A virus pose a serious threat to public health. It is sufficient to mention human cases of highly pathogenic H5N1 and H7N9 avian influenza in Asia and Europe or swine-origin H3N2v outbreaks in North America [35,36,37,38]. The lack of resistance to such strains in the human population is caused by continuous genetic variation in the virus, which is based on phenomena such as reassortment, antigenic shift, or antigenic drift [39]. Prophylactic vaccination against currently circulating human strains shows high efficiency, but due to the variability of surface antigens, hemagglutinin (HA), and neuraminidase (NA), the treatment must be repeated every year with a new vaccine, the composition of which is determined on the basis of antigenic drift. Furthermore, in the case of the emergence of an entirely new strain resulting from reassortment, seasonal vaccines will most likely prove ineffective [40].

Reassortment of genetic segments during coinfection of one organism with strains from different hosts could serve as a potential source of new, dangerous strains of influenza A virus and may lead to overcoming species barriers and adaptation of the virus to new hosts. The majority of avian influenza viruses originating from natural reservoirs cannot cause serious diseases in humans. However, some zoonotic strains that acquired the possibility of infection in the human population caused serious disease and, in some cases, fatalities [41,42,43,44].

During the pandemic, the new A (H1N1) pdm09 strain was also detected in animals, alarming farmers and organizations involved in public and animal health and pushing them to identify the origin of the pandemic strain [45]. It was found that it was a reassortant combined with four different strains of the virus. It contained a combination of genes from the “human” influenza virus, the “swine” influenza virus from North America, the Asian “swine” influenza virus of avian origin, and the “avian” influenza virus from North America. This reassortment of influenza virus strains has never been recorded previously [46]. In a short time, the new pandemic strain was responsible for the majority of influenza cases worldwide. Before the beginning of the post-pandemic period, the seasonal H1N1 circulating among humans was almost entirely displaced. Simultaneously, A(H1N1)pdm09 infected swine and spread to pig populations in Europe [47,48]. There has been evidence of specific strain circulation between humans and swine, as a high degree of homology was observed between the sequences of isolates collected during local epidemics from both hosts [49,50,51]. Infection of pigs with the “human” virus poses a serious risk of the emergence of a new virus strain capable of efficient transmission between humans [3]. Most likely, this scenario led to an outbreak of H3N2v in the U.S. in 2011 and 2012. The newly emerged strain was a swine reassortant that acquired gene segments from the swine H3N2 triple reassortant virus, circulating in the U.S. in 1998, and an *M* gene from pandemic H1N1 [52]. Previous contact with pigs was the main etiology of the infections, but some cases of human-to-human transmission have also been reported, likely due to the presence of the M gene from the pandemic strain. [53]. A similar event occurred independently in Europe in 2012. A triple reassortant A/swine/Spain/28778/2012 was isolated in Spain, and similar to the North American strain, it contained external glycoprotein genes from H3N2, in this case from the Gent/84-like strain, an almost complete IGC from the Eurasian avian lineage, and the M gene from the H1N1pdm09-like strain. This shows how the independent evolution of the virus on two continents can lead to the emergence of a strain with similar epidemiological features [22]. Since the end of the 2009 pandemic, cases of human infection with the swine-origin influenza virus have been reported in numerous locations in Europe and Asia, some of which are fatal [25,27,33,54,55,56,57]. Conversely, reverse zoonosis cases have been known for a long time. Some of them are well documented as pivotal changes in swine influenza diversity [58]; for example, the introduction of the H3N2 “Port Chalmer’s like” virus into swine populations in Europe or the sustained presence of the A(H1N1)pdm09 virus among herds in the US and Europe [49,59,60]. Furthermore, there is some evidence supporting the fact that human-to-swine transmission frequency exceeds the reverse scenario, and the human–swine interface must be perceived as bidirectional to fully understand complex influenza A ecology and evolution [33].

The initiative to assess the epidemiological situation among swine influenza viruses in Europe gained momentum after the H1N1pdm09 lineage became well-established in the pig population. Together with evolving sequencing and bioinformatics methods, new insights into the origin of circulating strains were made possible. Moreno et al. (2013) [61] and Trebbien et al. (2013) [16] show different evolutionary trends and reassortation events in Italy and Denmark when analyzing samples from the 2000–2012 period. Although focused on a specific country and with a small number of samples, they provided a good perspective on the epidemiological situation of swIAVs in the pre-pandemic years [16,61]. To date, the largest and most diverse surveillance programs have been carried out by the European Surveillance Network for Influenza in Pigs (ESNIP-3) and Watson et al. (2015) [22], followed by a study described by Henritzi et al. (2020) [23]. The first program analyzed 290 isolates collected from 14 European countries between 2009 and 2013. They identified 23 distinct genotypes (A-W) and proposed a classification system to distinguish between the main lineages and reassortant strains. At the animal level, H1avN1 (genotype A) was the most common strain (29%), followed by H3N2 (genotype B) (13%), H1N1pdm09 (genotype P) (12%), and H1huN2 (genotype C) (9%). Evidence showed a significant level of replacement of the endemic Eurasian avian viruses with H1N1pdm09-like strains and a high number of reassortation events, leading to high genotype diversity among European countries [22]. The latter program was based on over 18,000 samples collected from approximately 2500 farms in 17 countries between 2015 and 2018. The study showed that 56.6% of swine holdings were infected with viruses belonging to the four major lineages. Twelve distinct HA/NA combinations were added to those reported by Watson et al. (2015) [22]. At the farm level, 39.2% of isolates belonged to the H1avN1av subtype, which is the most dominant subtype. The H1avN2 reassortant had a prevalence of 12.6% with H1huN2 at almost the same level (11.4%), and H1N1pdm09 accounted for 4.2% of cases. H3N2 subtype was more rarely detected (3.2%) and H3 strains also showed the lowest antigenic diversity, especially in comparison with H1pdm subtypes showing complex phylogenetic patterns and high evolution dynamics [23].

Our previous study carried out on virus isolates obtained from pigs in Poland revealed coinfection of animals with several different genetic variants of influenza A virus, some associated with human influenza A strains, most likely A/California/07/2009-like strains [62]. There is evidence that the number of pigs in Poland, which are prone to the risk of mixed infections, may constitute a reservoir of potentially dangerous strains of influenza A virus, resulting from genetic reassortment. Our findings were supported by a study described in Czyzewska-Dors et al. (2017) [29], where over 5900 samples from 145 farms in Poland were analyzed to assess the seroprevalence of swIAV in farrow-to-finish pig herds and in almost 18% of herds antibodies against all four major lineages (H1N1, H1N2, H3N2, and H1N1pdm09) were detected [29].

The data presented here are part of a study in which we analyzed over 370 samples taken from 11 swine herds in northwestern Poland. We were able to confirm the infection in 112 samples from 5 of the 11 herds, and we obtained 93 full genome sequences. All strains belonged to either A/H1N2 (63.4%) or A/H1N1(36.6%) swine influenza subtypes. Molecular subtyping and phylogenetic analysis showed that H1N2 strains found in three herds (27.3%) represented the T genotype, a reassortant strain carrying the H1av gene, the N2 gene related to A/swine/Gent/1/1984, and IGC of pandemic origin. All H1N1 strains found in one herd (9.1%) represented the P genotype, where all the genetic segments were derived from H1N1pdm09. Multiple alignments of all segment sequences revealed five distinct genetic variants of H1N2 and H1N1 isolates (three and two, respectively).

To discuss the genotype–phenotype relationship of the T (H1avN2g, GI-GIII) and P (H1pdmN1pdm, GIV-GV) genotypes, we checked whether the genetic variances of HA, NA, and/or PB2 in these viral isolates lead to amino acid substitutions that may facilitate enhanced replication in humans. All analyzed strains lacked substitutions in the PB2 gene, such as E627K, D701N, T271A, E158G, D309N, T339M, or S714R, which have been identified as important markers for higher pathogenicity of influenza viruses in mammals [63,64,65]. However, they all have an arginine at position 591, which can compensate for the lack of the E627K mutation and enhance viral replication of pandemic H1N1 in mammals [66]. Interestingly, H1N2 genotypes (GI-GIII) have the PB2 amino acid 249G, which is likely involved in mammalian adaptation mechanisms, since it can be found in mouse-adapted strains [64]. Both T and P genotypes from this study harbored E190D/G225E/D amino acid substitutions in the receptor binding site of HA that alter receptor binding specificity towards human-type receptors, which is not surprising in swine viruses since those adaptations were widespread shortly after the 2009 pandemic [53,67,68]. The difference between the T and P genotypes in this case is that the former has glutamic acid and the latter has aspartic acid instead of glycine at position 225. None of the analyzed strains possessed the K163E and D222G substitutions associated with fatal and severe outcomes of pandemic influenza [69] or L425M, which is known to enhance the replication and pathogenicity of rH1N1 in mice. H1pdmN1pdm isolates do not exhibit oseltamivir resistance conferred by the H275Y substitution in N1, and H1avN2g isolates lack the N2 amino acid 151G, which is responsible for HA-independent receptor binding [70,71].

The general number of infections in farms (45.5%) and animals (29.8%) was very similar to the latest large-scale European survey conducted by Henritzi et al. (2020) [23] (56.6% and 30.5%, respectively), although when we look at particular genotype prevalence, surprisingly we did not find any H1avN1 subtype, dominant in Europe in recent years. In contrast, the incidence of H1avN2 in our study was almost the same (27.3% to 25%), and H1N1pdm09 was slightly lower (9.1% to 12.5%). Taking into account the fact that IGCs from all strains described in this study are of pandemic origin, the lower incidence of H1N1pdm09 may be the result of the high rate of external glycoproteins segments exchange during reassortment events. The most recent data concerning the Polish swIAV are those described by Vereecke et al. (2023) [72]. Nineteen isolates from 14 Polish farms collected between 2017 and 2020 were analyzed and classified as genotypes T (58.3%), U (25%), P (16.7%), and A (7.1%). The incidence levels of particular subtypes differ from our findings but genotypes T and P also constitute the majority of the analyzed strains, and the genotype T/P ratio is comparable (33.4% to 28.6%). We must also consider that these two studies were conducted on a small number of isolates, and without further investigation and country-wide sampling on a much larger scale, we cannot properly assess the genetic diversity of swIAVs circulating among herds in Poland; therefore, there is a need for a broad surveillance program coordinated by veterinary services.

## 5. Conclusions

The pandemic potential of zoonotic strains cannot be underestimated, and public health measures must be implemented to prevent and manage infections in human and animal ecosystems, particularly in farm animals.

There are very few epidemiological studies involving the full genetic characterization of strains and detection of co-infections with influenza A virus in Poland that have been carried out among livestock populations. In the interest of public health, we should assess the risk within the human–animal interface, as it may serve as a reservoir of potentially dangerous, new genetic variants of the influenza A virus.

## Figures and Tables

**Table 1:** Summary of sample collection, molecular subtyping, HA clade, and NA lineage classification. Percentage (%) of positive samples is given in brackets. “No data available” (n/d) description indicates that no virus was isolated from the 03KA location samples. Subtyping performed as in Chiapponi et al. (2021), HA clade classification based on tools described by Anderson et al. (2016), and NA lineage based on Watson et al. (2015). N2g = A/swine/Gent/1/1984-like, pdm = N1pdm09.

Localization	Time of Collection	Number of Samples	Number of Confirmed Influenza Infections	Subtype	HA Clade	NA Lineage
01KO	05/2017	35	0	-	-	-
02SY	07/2017	45	24 (53%)	H1N2	1C.2	N2g
03KA	05/2018	30	16 (53%)	n/d	n/d	n/d
04BA	10/2018	40	19 (47.5%)	H1N1	1A.3.3.2	pdm
05PA	11/2018	20	0	-	-	-
06KU	03/2019	40	22 (55%)	H1N2	1C.2	N2g
07RA	03/2019	20	16 (80%)	H1N2	1C.2	N2g
08KO	06/2019	21	0	-	-	-
04BA	07/2019	35	15 (42.8%)	H1N1	1A.3.3.2	pdm
09WI	09/2019	25	0	-	-	-
10MI	10/2019	30	0	-	-	-
11GI	10/2019	35	0	-	-	-
TOTAL		376	112 (29.8%)			

**Table 2:** List of strains corresponding to five genetic variants present in all sequenced samples. Individual segment sequences of complete genomes were deposited in GISAID EpiFlu™. Database under accession numbers provided.

Genetic Variant	Corresponding Strain	Accession Number
G I	A/swine/Poland/GI-S01/2019(H1N2)	EPI_ISL_17952486
G II	A/swine/Poland/GII-S02/2017(H1N2)	EPI_ISL_17952487
G III	A/swine/Poland/GIII-S38/2019(H1N2)	EPI_ISL_17952488
G IV	A/swine/Poland/GIV-S21/2018(H1N1)	EPI_ISL_17952489
G V	A/swine/Poland/GV-S23/2019(H1N1)	EPI_ISL_17952490

**Table 3:** List of genotypes found in all 93 analyzed samples and relative percentages (%). Genotyping according to Watson et al. (2015). N2g = A/swine/Gent/1/1984-like, pdm = N1pdm09. H1 clade classified according to Anderson et al. (2015).

Subtype	Genotype	HA	NA	PB2	PB1	PA	NP	M	NS	% of Analyzed Samples
H1N1	P	1A.3.3.2	pdm	pdm	pdm	pdm	pdm	pdm	pdm	36.6%
H1N2	T	1C.2	N2g	pdm	pdm	pdm	pdm	pdm	pdm	63.4%

## Data Availability

The data presented in this study are available upon request.

## Supplementary Materials

The following supporting information can be downloaded at: https://www.mdpi.com/article/10.3390/v15091893/s1 [63], Table S1: Reference strains dataset and accession numbers.

## References

[1] Yoon S.-W., Webby R.J., Webster R.G., Compans R.W., Oldstone M.B.A. (2014). Evolution and Ecology of Influenza A Viruses. Influenza Pathogenesis and Control—Volume I.

[2] Suarez D.L. (2016). Influenza A Virus. Animal Influenza.

[3] Goneau L.W., Mehta K., Wong J., L’Huillier A.G., Gubbay J.B. (2018). Zoonotic Influenza and Human Health—Part 1: Virology and Epidemiology of Zoonotic Influenzas. Curr. Infect. Dis. Rep..

[4] Mänz B., Schwemmle M., Brunotte L. (2013). Adaptation of Avian Influenza A Virus Polymerase in Mammals To Overcome the Host Species Barrier. J. Virol..

[5] Linster M., van Boheemen S., de Graaf M., Schrauwen E.J.A., Lexmond P., Mänz B., Bestebroer T.M., Baumann J., van Riel D., Rimmelzwaan G.F. (2014). Identification, Characterization, and Natural Selection of Mutations Driving Airborne Transmission of A/H5N1 Virus. Cell.

[6] Dubois J., Terrier O., Rosa-Calatrava M. (2014). Influenza Viruses and MRNA Splicing: Doing More with Less. mBio.

[7] Reperant L.A., Grenfell B.T., Osterhaus A.D.M.E. (2015). Quantifying the Risk of Pandemic Influenza Virus Evolution by Mutation and Re-Assortment. Vaccine.

[8] Feldblyum T.V., Segal D.M. (2015). Seasonal and Pandemic Influenza Surveillance and Disease Severity. Glob. Virol. I—Identifying Investig. Viral Dis..

[9] Petrova V.N. (2018). The Evolution of Seasonal Influenza Viruses. Nat. Rev. Microbiol..

[10] Forrest H.L., Webster R.G. (2010). Perspectives on Influenza Evolution and the Role of Research. Anim. Health Res. Rev..

[11] Scholtissek C. (1990). Pigs as ‘Mixing Vessels’ for the Creation of New Pandemic Influenza A Viruses. Med. Princ. Pract..

[12] Krumbholz A., Lange J., Sauerbrei A., Groth M., Platzer M., Kanrai P., Pleschka S., Scholtissek C., Büttner M., Dürrwald R. (2014). Origin of the European Avian-like Swine Influenza Viruses. J. Gen. Virol..

[13] Campitelli L., Donatelli I., Foni E., Castrucci M.R., Fabiani C., Kawaoka Y., Krauss S., Webster R.G. (1997). Continued Evolution of H1N1 and H3N2 Influenza Viruses in Pigs in Italy. Virology.

[14] Brown I.H., Harris P.A., McCauley J.W., Alexander D.J. (1998). Multiple Genetic Reassortment of Avian and Human Influenza A Viruses in European Pigs, Resulting in the Emergence of an H1N2 Virus of Novel Genotype. J. Gen. Virol..

[15] Mena I., Nelson M.I., Quezada-Monroy F., Dutta J., Cortes-Fernández R., Lara-Puente J.H., Castro-Peralta F., Cunha L.F., Trovão N.S., Lozano-Dubernard B. (2016). Origins of the 2009 H1N1 Influenza Pandemic in Swine in Mexico. eLife.

[16] Trebbien R., Bragstad K., Larsen L.E., Nielsen J., Bøtner A., Heegaard P.M.H., Fomsgaard A., Viuff B., Hjulsager C.K. (2013). Genetic and Biological Characterisation of an Avian-like H1N2 Swine Influenza Virus Generated by Reassortment of Circulating Avian-Like H1N1 and H3N2 Subtypes in Denmark. Virol. J..

[17] Krog J.S., Hjulsager C.K., Larsen M.A., Larsen L.E. (2017). Triple-reassortant Influenza A Virus with H3 of Human Seasonal Origin, NA of Swine Origin, and Internal A(H1N1) Pandemic 2009 Genes Is Established in Danish Pigs. Influenza Other Respir Viruses.

[18] Zell R., Groth M., Krumbholz A., Lange J., Philipps A., Dürrwald R. (2020). Displacement of the Gent/1999 Human-like Swine H1N2 Influenza A Virus Lineage by Novel H1N2 Reassortants in Germany. Arch. Virol..

[19] Chiapponi C., Prosperi A., Moreno A., Baioni L., Faccini S., Manfredi R., Zanni I., Gabbi V., Calanchi I., Fusaro A. (2021). Genetic Variability among Swine Influenza Viruses in Italy: Data Analysis of the Period 2017–2020. Viruses.

[20] Chepkwony S., Parys A., Vandoorn E., Stadejek W., Xie J., King J., Graaf A., Pohlmann A., Beer M., Harder T. (2021). Genetic and Antigenic Evolution of H1 Swine Influenza A Viruses Isolated in Belgium and the Netherlands from 2014 through 2019. Sci. Rep..

[21] Chastagner A., Hervé S., Bonin E., Quéguiner S., Hirchaud E., Henritzi D., Béven V., Gorin S., Barbier N., Blanchard Y. (2018). Spatiotemporal Distribution and Evolution of the A/H1N1 2009 Pandemic Influenza Virus in Pigs in France from 2009 to 2017: Identification of a Potential Swine-Specific Lineage. J. Virol..

[22] Watson S.J., Langat P., Reid S.M., Lam T.T.-Y., Cotten M., Kelly M., Van Reeth K., Qiu Y., Simon G., Bonin E. (2015). Molecular Epidemiology and Evolution of Influenza Viruses Circulating within European Swine between 2009 and 2013. J. Virol..

[23] Henritzi D., Petric P.P., Lewis N.S., Graaf A., Pessia A., Starick E., Breithaupt A., Strebelow G., Luttermann C., Parker L.M.K. (2020). Surveillance of European Domestic Pig Populations Identifies an Emerging Reservoir of Potentially Zoonotic Swine Influenza A Viruses. Cell Host Microbe.

[24] Anderson T.K., Macken C.A., Lewis N.S., Scheuermann R.H., Van Reeth K., Brown I.H., Swenson S.L., Simon G., Saito T., Berhane Y. (2016). A Phylogeny-Based Global Nomenclature System and Automated Annotation Tool for H1 Hemagglutinin Genes from Swine Influenza A Viruses. mSphere.

[25] Zell R., Scholtissek C., Ludwig S. (2013). Genetics, Evolution, and the Zoonotic Capacity of European Swine Influenza Viruses. Curr. Top. Microbiol. Immunol..

[26] Novel Swine-Origin Influenza A (H1N1) Virus Investigation Team (2009). Emergence of a Novel Swine-Origin Influenza A (H1N1) Virus in Humans. N. Engl. J. Med..

[27] Sun H., Xiao Y., Liu J., Wang D., Li F., Wang C., Li C., Zhu J., Song J., Sun H. (2020). Prevalent Eurasian Avian-like H1N1 Swine Influenza Virus with 2009 Pandemic Viral Genes Facilitating Human Infection. Porc. Natl. Acad. Sci. USA.

[28] Er J.C., Lium B., Framstad T. (2020). Antibodies of Influenza A(H1N1)Pdm09 Virus in Pigs’ Sera Cross-React with Other Influenza A Virus Subtypes. A Retrospective Epidemiological Interpretation of Norway’s Serosurveillance Data from 2009–2017. Epidemiol. Infect..

[29] Czyżewska-Dors E., Dors A., Kwit K., Pejsak Z., Pomorska-Mól M. (2017). Serological Survey of the Influenza a Virus in Polish Farrow-to-Finish Pig Herds in 2011–2015. J. Vet. Res..

[30] Zoonotic Influenza- Annual Epidemiological Report for 2014. https://www.ecdc.europa.eu/en/publications-data/zoonotic-influenza-annual-epidemiological-report-2014.

[31] Threat Assessment Brief: Eurasian Avian-like A(H1N1) Swine Influenza Viruses. https://www.ecdc.europa.eu/en/publications-data/threat-assessment-brief-eurasian-avian-ah1n1-swine-influenza-viruses.

[32] Jhung M.A., Epperson S., Biggerstaff M., Allen D., Balish A., Barnes N., Beaudoin A., Berman L., Bidol S., Blanton L. (2013). Outbreak of Variant Influenza A(H3N2) Virus in the United States. Clin. Infect. Dis..

[33] Nelson M.I., Vincent A.L. (2015). Reverse Zoonosis of Influenza to Swine: New Perspectives on the Human-Animal Interface. Trends Microbiol..

[34] Krammer F., Palese P. (2015). Advances in the Development of Influenza Virus Vaccines. Nat. Rev. Drug Discov..

[35] Sutton T.C. (2018). The Pandemic Threat of Emerging H5 and H7 Avian Influenza Viruses. Viruses.

[36] Soh Y.S., Moncla L.H., Eguia R., Bedford T., Bloom J.D. (2019). Comprehensive Mapping of Adaptation of the Avian Influenza Polymerase Protein PB2 to Humans. eLife.

[37] Wong K.K., Gambhir M., Finelli L., Swerdlow D.L., Ostroff S., Reed C. (2013). Transmissibility of Variant Influenza From Swine to Humans: A Modeling Approach. Clin. Infect. Dis..

[38] Cauchemez S., Epperson S., Biggerstaff M., Swerdlow D., Finelli L., Ferguson N.M. (2013). Using Routine Surveillance Data to Estimate the Epidemic Potential of Emerging Zoonoses: Application to the Emergence of US Swine Origin Influenza A H3N2v Virus. PLoS Med..

[39] White M.C., Lowen A.C. (2018). Implications of Segment Mismatch for Influenza A Virus Evolution. J. Gen. Virol..

[40] Tricco A.C., Chit A., Soobiah C., Hallett D., Meier G., Chen M.H., Tashkandi M., Bauch C.T., Loeb M. (2013). Comparing Influenza Vaccine Efficacy against Mismatched and Matched Strains: A Systematic Review and Meta-Analysis. BMC Med..

[41] Wright P.F., Neumann G., Kawaoka Y. (2013). Orthomyxoviruses. Fields Virology.

[42] Lam T.T.-Y., Wang J., Shen Y., Zhou B., Duan L., Cheung C.-L., Ma C., Lycett S.J., Leung C.Y.-H., Chen X. (2013). The Genesis and Source of the H7N9 Influenza Viruses Causing Human Infections in China. Nature.

[43] Smith G.J.D., Bahl J., Vijaykrishna D., Zhang J., Poon L.L.M., Chen H., Webster R.G., Peiris J.S.M., Guan Y. (2009). Dating the Emergence of Pandemic Influenza Viruses. Porc. Natl. Acad. Sci. USA.

[44] Guan Y., Shortridge K.F., Krauss S., Webster R.G. (1999). Molecular Characterization of H9N2 Influenza Viruses: Were They the Donors of the “Internal” Genes of H5N1 Viruses in Hong Kong?. Proc. Natl. Acad. Sci. USA.

[45] Nelson M.I., Gramer M.R., Vincent A.L., Holmes E.C. (2012). Global Transmission of Influenza Viruses from Humans to Swine. J. Gen. Virol..

[46] Garten R.J., Davis C.T., Russell C.A., Shu B., Lindstrom S., Balish A., Sessions W.M., Xu X., Skepner E., Deyde V. (2009). Antigenic and Genetic Characteristics of Swine-Origin 2009 A(H1N1) Influenza Viruses Circulating in Humans. Science.

[47] Forgie S.E., Keenliside J., Wilkinson C., Webby R., Lu P., Sorensen O., Fonseca K., Barman S., Rubrum A., Stigger E. (2011). Swine Outbreak of Pandemic Influenza A Virus on a Canadian Research Farm Supports Human-to-Swine Transmission. Clin. Infect. Dis. Off. Publ. Infect. Dis. Soc. Am..

[48] Simon G., Larsen L.E., Dürrwald R., Foni E., Harder T., Van Reeth K., Markowska-Daniel I., Reid S.M., Dan A., Maldonado J. (2014). European Surveillance Network for Influenza in Pigs: Surveillance Programs, Diagnostic Tools and Swine Influenza Virus Subtypes Identified in 14 European Countries from 2010 to 2013. PLoS ONE.

[49] Brown I.H., Richt J.A., Webby R.J. (2013). History and Epidemiology of Swine Influenza in Europe. Swine Influenza.

[50] Hofshagen M., Gjerset B., Er C., Tarpai A., Brun E., Dannevig B., Bruheim T., Fostad I.G., Iversen B., Hungnes O. (2009). Pandemic Influenza A(H1N1)v: Human to Pig Transmission in Norway?. Eurosurveillance.

[51] Grøntvedt C.A., Er C., Gjerset B., Germundsson A., Framstad T., Brun E., Jørgensen A., Lium B. (2011). Clinical Impact of Infection with Pandemic Influenza (H1N1) 2009 Virus in Naïve Nucleus and Multiplier Pig Herds in Norway. Influenza Res. Treat..

[52] Ducatez M.F., Hause B., Stigger-Rosser E., Darnell D., Corzo C., Juleen K., Simonson R., Brockwell-Staats C., Rubrum A., Wang D. (2011). Multiple Reassortment between Pandemic (H1N1) 2009 and Endemic Influenza Viruses in Pigs, United States. Emerg. Infect. Dis..

[53] Rajão D.S., Walia R.R., Campbell B., Gauger P.C., Janas-Martindale A., Killian M.L., Vincent A.L. (2017). Reassortment between Swine H3N2 and 2009 Pandemic H1N1 in the United States Resulted in Influenza A Viruses with Diverse Genetic Constellations with Variable Virulence in Pigs. J. Virol..

[54] Rovida F., Piralla A., Marzani F.C., Moreno A., Campanini G., Mojoli F., Pozzi M., Girello A., Chiapponi C., Vezzoli F. (2017). Swine influenza A (H1N1) Virus (SIV) Infection Requiring Extracorporeal Life Support in an Immunocompetent Adult Patient with Indirect Exposure to Pigs, Italy, October 2016. Eurosurveillance.

[55] Li X., Guo L., Liu C., Cheng Y., Kong M., Yang L., Zhuang Z., Liu J., Zou M., Dong X. (2019). Human Infection with a Novel Reassortant Eurasian-Avian Lineage Swine H1N1 Virus in Northern China. Emerg. Microbes Infect..

[56] Fraaij P.L.A., Wildschut E.D., Houmes R.J., Swaan C.M., Hoebe C.J., de Jonge H.C.C., Tolsma P., de Kleer I., Pas S.D., Oude Munnink B.B. (2016). Severe Acute Respiratory Infection Caused by Swine Influenza Virus in a Child Necessitating Extracorporeal Membrane Oxygenation (ECMO), the Netherlands, October 2016. Eurosurveillance.

[57] Adlhoch C., Penttinen P. (2017). Letter to the Editor: Just a Coincidence? Two Severe Human Cases Due to Swine Influenza (SIV) A(H1N1)v in Europe, October 2016. Eurosurveillance.

[58] Mostafa A., Abdelwhab E.M., Mettenleiter T.C., Pleschka S. (2018). Zoonotic Potential of Influenza A Viruses: A Comprehensive Overview. Viruses.

[59] Zhu H., Webby R., Lam T.T.Y., Smith D.K., Peiris J.S.M., Guan Y. (2013). History of Swine Influenza Viruses in Asia. Curr. Top. Microbiol. Immunol..

[60] Lorusso A., Vincent A.L., Gramer M.E., Lager K.M., Ciacci-Zanella J.R. (2013). Contemporary Epidemiology of North American Lineage Triple Reassortant Influenza A Viruses in Pigs. Curr. Top. Microbiol. Immunol..

[61] Moreno A., Gabanelli E., Sozzi E., Lelli D., Chiapponi C., Ciccozzi M., Zehender G., Cordioli P. (2013). Different Evolutionary Trends of Swine H1N2 Influenza Viruses in Italy Compared to European Viruses. Vet. Res..

[62] Lepek K., Pajak B., Rabalski L., Urbaniak K., Kucharczyk K., Markowska-Daniel I., Szewczyk B. (2015). Analysis of Coinfections with A/H1N1 Strain Variants among Pigs in Poland by Multitemperature Single-Strand Conformational Polymorphism. BioMed. Res. Int..

[63] Hu J., Hu Z., Wei Y., Zhang M., Wang S., Tong Q., Sun H., Pu J., Liu J., Sun Y. (2022). Mutations in PB2 and HA Are Crucial for the Increased Virulence and Transmissibility of H1N1 Swine Influenza Virus in Mammalian Models. Vet. Microbiol..

[64] Yamaji H. (2014). Suitability and Perspectives on Using Recombinant Insect Cells for the Production of Virus-like Particles. Appl. Microbiol. Biotechnol..

[65] Czudai-Matwich V., Otte A., Matrosovich M., Gabriel G., Klenk H.-D. (2014). PB2 Mutations D701N and S714R Promote Adaptation of an Influenza H5N1 Virus to a Mammalian Host. J. Virol..

[66] Yamada S., Hatta M., Staker B.L., Watanabe S., Imai M., Shinya K., Sakai-Tagawa Y., Ito M., Ozawa M., Watanabe T. (2010). Biological and Structural Characterization of a Host-Adapting Amino Acid in Influenza Virus. PLoS Pathog..

[67] Thompson A.J., Paulson J.C. (2021). Adaptation of Influenza Viruses to Human Airway Receptors. J. Biol. Chem..

[68] Hennig C., Graaf A., Petric P.P., Graf L., Schwemmle M., Beer M., Harder T. (2022). Are Pigs Overestimated as a Source of Zoonotic Influenza Viruses?. Porc. Health Manag..

[69] Wedde M., Wählisch S., Wolff T., Schweiger B. (2013). Predominance of HA-222D/G Polymorphism in Influenza A(H1N1)Pdm09 Viruses Associated with Fatal and Severe Outcomes Recently Circulating in Germany. PLoS ONE.

[70] Zhu X., McBride R., Nycholat C.M., Yu W., Paulson J.C., Wilson I.A. (2012). Influenza Virus Neuraminidases with Reduced Enzymatic Activity That Avidly Bind Sialic Acid Receptors. J. Virol..

[71] Lin Y.P., Gregory V., Collins P., Kloess J., Wharton S., Cattle N., Lackenby A., Daniels R., Hay A. (2010). Neuraminidase Receptor Binding Variants of Human Influenza A(H3N2) Viruses Resulting from Substitution of Aspartic Acid 151 in the Catalytic Site: A Role in Virus Attachment?. J. Virol..

[72] Vereecke N., Woźniak A., Pauwels M., Coppens S., Nauwynck H., Cybulski P., Theuns S., Stadejek T. (2023). Successful Whole Genome Nanopore Sequencing of Swine Influenza A Virus (SwIAV) Directly from Oral Fluids Collected in Polish Pig Herds. Viruses.

